# The Efficacy of Diagnostic Plaster Models in Orthodontic Diagnosis and Treatment Planning

**DOI:** 10.3390/diagnostics14192124

**Published:** 2024-09-25

**Authors:** Abdullazez Almudhi, Iman Almohammad, Sara Alswayyed, Elzahraa Eldwakhly, Sarah Almugairin

**Affiliations:** 1 Department of Pediatric Dentistry and Orthodontics, College of Dentistry, King Saud University, Riyadh 11545, Saudi Arabia; 2 College of Dentistry, King Saud University, Riyadh 11545, Saudi Arabia; 3 Department of Clinical Dental Sciences, College of Dentistry, Princess Nourah bint Abdulrahman University, Riyadh 11671, Saudi Arabia; 4 Department of Preventive Dental Sciences, College of Dentistry, Princess Nourah bint Abdulrahman University, Riyadh 11671, Saudi Arabia

**Keywords:** orthodontic diagnosis, treatment planning, plaster models, digital records, orthodontic records

## Abstract

Background: The growing integration of digital technologies in orthodontics is shifting the orthodontic diagnosis and recordkeeping paradigm, replacing conventional plaster models with intraoral scanning and 3D photography. This study investigated the impact of orthodontic plaster models on orthodontic diagnosis and treatment planning decisions by orthodontists. Methods: Thirty-three orthodontists assessed six patients’ records with different malocclusion cases. Each case was assessed twice by each orthodontist evaluating a case: the first evaluation with digital records without diagnostic casts and the second evaluation with the added diagnostic orthodontic plaster model. Diagnostic and treatment plan decisions for each malocclusion case were compared with and without the aid of the diagnostic orthodontic plaster models to assess the plaster model’s impact on the treatment plan’s soundness. Results: Statistically insignificant differences were found for the diagnoses and treatment plans with or without the aid of orthodontic plaster models. Intraclass correlation coefficients revealed agreement among orthodontists for both evaluated situations (0.685; *p* < 0.0001). Plaster models were found to significantly influence orthodontists’ decisions about the need for surgical intervention (*p* = 0.026), but they did not significantly impact the overall malocclusion diagnostic classification nor the decision regarding the extent of treatment, whether comprehensive or limited (*p* = 0.146) and extraction versus non-extraction approaches (*p* = 0.266). Conclusions: These findings support the idea that digital record alternatives may be viable for orthodontic recordkeeping purposes. Within the limitations of this study, it can be concluded that the presence or absence of orthodontic plaster models does not significantly impact the orthodontic diagnosis or treatment planning process.

## 1. Introduction

Recently, digital technologies have been massively integrated across the dental healthcare industry [1]. Digitization is applied in orthodontics to digitize patient information and diagnostic data [2,3]. The conventional dental plaster models are recognized for their ease of fabrication, affordability, and accuracy and are considered the gold standard for diagnosis, treatment planning, and occlusal assessment in orthodontics [4]. Nonetheless, it also comes with significant limitations, such as requiring significant storage space, being prone to breakage, and being inefficient in data retrieval and transfer [5,6]. These drawbacks promoted digital transformation in orthodontics, which promises improved efficiency, reduced costs, and enhanced patient care [7,8].

Digital orthodontic record systems combined with digital radiography, photography, intraoral scanning, and the replacement of physical plaster models with digital records are all means of digitizing the orthodontic diagnostic process [1,9].

The digital diagnostic recordkeeping process has been documented as a practical alternative to traditional recordkeeping with several advantages for orthodontists and patients [10]. Firstly, it improves efficiency in managing and sharing patient records with dental labs without packing and sending impressions and eliminates all infection control concerns [11]. Secondly, integrating digital diagnostic records provides a more holistic view of patients’ oral structures with improved visualization and precise measurements, leading to more accurate diagnoses [12]. Thirdly, digital impression-making eliminates the discomfort of physical impressions by reducing the gag reflex and providing more comfort for patients [2,13].

Traditional diagnosis in orthodontics involves a comprehensive medical and dental history review, thorough clinical examination, and patient records such as radiographs, study models, and photographs [14]. Radiographic examinations play an important role in orthodontists’ initial diagnosis and treatment planning by disclosing information not apparent with clinical examination [15]. Conventional two-dimensional radiographs (panoramic, periapical, occlusal, and lateral cephalograms) have inherent limitations such as magnification, distortion, and superimposition [16]. This led to the adoption of digital imaging and cone beam computed tomography (CBCT), which offers a 3D view of the dentofacial structures and provides a more comprehensive view of complex cases [16,17].

Digital records compiled through intraoral scanning and computer-aided design and manufacturing (CAD/CAM) technologies greatly improve the workflow and efficiency of orthodontic practice [18]. The ability to manipulate, rotate, and magnify digital records on screen provides a more comprehensive visualization of the dentition and surrounding structures, contributing to improved diagnostic accuracy. Still, digital records present challenges, such as data loss, high equipment costs, and reliance on third-party systems [19].

Several studies documented that three-dimensional virtual digital study models are clinically acceptable alternatives to plaster study models in treatment planning and decision-making for malocclusion patients due to linear measurements’ reliability, validity, and reproducibility [2,20,21]

Insignificant differences were reported for treatment planning options using digital versus traditional plaster models [22]. Several studies examined the reliability of treatment planning decisions made according to conventional versus digital diagnostic aids and reported good reliability of digital records in treatment planning, thus providing confidence in the transition to digital technologies [14,23,24].

Due to the expanding use of digital diagnostic records in place of physical diagnostic models in orthodontic decision-making, it was significantly important to analyze the continued relevance of plaster models’ use in orthodontic diagnosis and treatment planning during the current digital era. This cross-sectional observational study aimed to evaluate the added value of incorporating physical plaster models compared to using only digital records during orthodontic diagnostics and treatment planning decisions. Specifically, it assessed how different diagnostic tools influenced clinical decision-making among orthodontists with varying experience levels. Key aspects of the study included evaluating the accuracy of diagnostic assessments, ensuring treatment planning consistency, and exploring whether the spatial information provided by physical models offered a perceptible advantage over digital models.

This study’s findings are expected to clarify whether adopting physical models leads to significant improvements in diagnosis and treatment outcomes or if digital records alone are sufficient for effective orthodontic planning.

## 2. Materials and Methods

### 2.1. Study Design

This is a cross-sectional comparative analysis where 33 specialized orthodontists evaluated two sets of orthodontic patient records: the first including digital treatment records (panoramic radiographs, cephalograms, intraoral scans, and intra/extraoral photographs) and the second set including the digital records plus a physical diagnostic plaster model.

### 2.2. Patient Record Selection

The records of six patients with different types of malocclusions were selected from the King Saud University Hospital orthodontic clinic. The inclusion criteria of patient records were as follows: (a) non-syndromic patients and (b) patients with complete standard conventional dental records (panoramic radiographs, cephalograms, intra- and extraoral photographs, and dental casts). Exclusion criteria of patient records included (a) syndromic patients and (b) patients with an incomplete set of standard conventional dental records. Before using their clinical records in the study, informed consent was obtained from the patients. Informed consent was also obtained from participating orthodontists before completing the questionnaire and starting the study. The Institutional Review Board at King Saud University, Saudi Arabia, approved this study by the “Approval of Research Project No. E-19-4223”.

### 2.3. Expert Panel

Thirty-three orthodontists with advanced orthodontic training (completed orthodontic residency and obtained a degree in orthodontics) participated in the study. Their knowledge and experience served as the benchmark against which the impact of plaster models on treatment planning decisions was assessed.

To minimize bias, cases were randomly chosen to represent different malocclusions. Examiner bias elimination was carried out by implementing blinding and randomization as well as ensuring that examiners were not the treating clinicians themselves. Accordingly, this study enhances its internal validity and minimizes bias, leading to more reliable and generalizable results.

### 2.4. Data Collection

Each orthodontist independently evaluated the six de-identified patient records presented in a randomized order. Only the gender and age of patients were disclosed. The evaluation process was carried out according to two different scenarios, separated by four weeks to lessen recall bias:

- Scenario 1: The patient records used were digital treatment records per se (panoramic radiographs, cephalograms, and intra- and extraoral photographs).
- Scenario 2: The patient records used were digital records plus diagnostic physical orthodontic plaster models. This design was used to determine whether the plaster model affected the orthodontist’s treatment plan for any of the evaluated malocclusion cases.

A four-week interval between the two evaluations was implemented to minimize subjective error, avoid eye fatigue, and ensure that independent assessments were performed by each of the orthodontists.

To standardize the evaluation process before starting the study, all 33 participating orthodontists received detailed instructions and participated in a standardized training session to ensure consistent and calibrated assessment methodologies. The training covered various aspects to maintain consistency in assessment methodologies. This included both theoretical components and practical sessions. The theoretical part encompassed guidelines on how to interpret and apply assessment criteria, while practical sessions involved hands-on exercises to familiarize the orthodontists with the tools and techniques used in the evaluation. The training was conducted in a face-to-face manner, which allowed for interactive sessions and immediate feedback. This format was chosen to facilitate clear communication and effective demonstration of procedures. During these sessions, orthodontists had the opportunity to ask questions and receive direct guidance on filling out the questionnaires accurately.

Data were collected using a structured questionnaire developed and validated to assess participating orthodontists’ discretion regarding the diagnosis and treatment planning of the presented malocclusion cases according to the two preset evaluation scenarios.

The questionnaire was divided into three primary sections:

- Orthodontist Demographics: Basic information about the orthodontist (e.g., gender and years of experience).
- Diagnostic Assessment: Orthodontists were asked to provide a comprehensive diagnosis based on the presented records.
- Treatment Planning: Orthodontists were required to outline a proposed treatment plan, specifying treatment modalities, timing, and whether diagnostic materials presented for each case in the two tested scenarios were sufficient to establish a valid treatment plan or if additional diagnostic information was required. This enabled the participating orthodontists to assess plaster models’ perceived benefits or limitations.

### 2.5. Data Analysis

Data were analyzed using SPSS 24.0 version (IBM Inc., Chicago, IL, USA) statistical software. Descriptive statistics (frequencies and percentages) were used to describe the participating orthodontists’ demographic variables. Intraclass correlation for the agreement was used to observe the agreement between orthodontists’ responses towards the diagnosis at two time points (without and with the diagnostic plaster model). McNemar’s chi-square test was used to compare the binary responses (agreement or disagreement) to the three treatment plan options between the two evaluation scenarios for each orthodontist. A *p*-value of ≤ 0.05 was used to report the statistical significance.

In this study, McNemar’s chi-square test was chosen to evaluate changes in diagnostic classifications and assess intra-examiner reliability as the data involve paired categorical comparisons. McNemar’s chi-square test aims to assess changes in classifications or decisions made by the same individuals under two different conditions. In this case, the orthodontists provided diagnoses for two scenarios: (a) using only digital records and (b) using digital records plus physical plaster models. McNemar’s test is suitable for dichotomous (binary) outcomes, such as whether a diagnosis changes (yes/no) between conditions. It evaluates whether there is a significant difference in the frequency of changes between these two conditions. McNemar’s test assumes that the data are paired (which they are, as each orthodontist provides two sets of diagnoses) and that the outcomes are dichotomous, meaning it looks at changes between two states.

### 2.6. Expected Outcome

This study design yielded 198 different diagnoses and treatment plans provided by the 33 orthodontists for the six assessed malocclusion cases for two evaluation scenarios (one with digital records per se and one with digital records plus diagnostic physical orthodontic plaster models) to clarify the impact of adding plaster models to orthodontists’ diagnostic and treatment planning decision-making process.

## 3. Results

**Figure 1:**
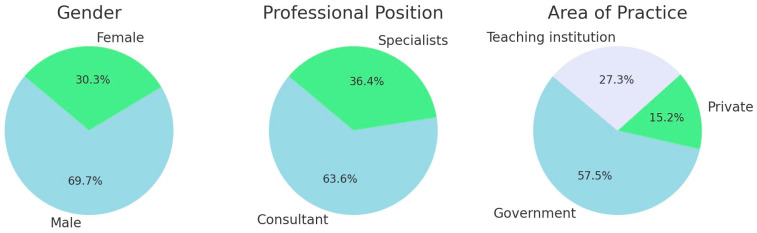
Pie chart representing distribution of participating orthodontists’ (*n* = 33) demographics.

Table 1 summarizes participating orthodontists’ demographics. The demographics of the participating orthodontists revealed the majority to be males (69.7%) and consultants (63.6%), and over half (50%) were employed in the government sector (Table 1; Figure 1).

### 3.1. Impact of Digital Records vs. Physical Diagnostic Plaster Models for Diagnosis of Orthodontic Malocclusion Classes

**Figure 2:**
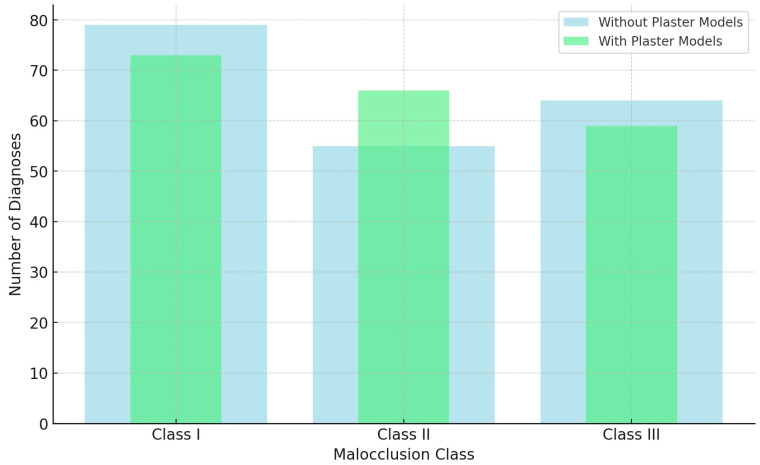
Impact of digital Records vs. diagnostic plaster models on diagnosis of orthodontic malocclusion.

Without the use of physical plaster models, the malocclusion diagnoses were distributed as follows: Class I malocclusion 79 times (39.3%), Class II 55 times (27.8%), and Class III 64 times (32.3%). When plaster models were incorporated, the distribution changed slightly: Class I was diagnosed 73 times (36.9%), Class II 66 times (33.3%), and Class III 59 times (29.8%). There were strong intra-observer agreements between diagnoses with both digital records and physical models (ICC = 0.685; *p* < 0.0001), indicating consistent diagnostic outcomes. This suggests that while plaster models may offer additional insights, they did not significantly alter malocclusion classification (Table 2; Figure 2).

### 3.2. Impact of Digital Records vs. Physical Diagnostic Plaster Models on Treatment Planning for Orthodontic Malocclusion Classes

#### 3.2.1. Treatment Plan One (Comprehensive Treatment Plan vs. Limited Treatment Plan)

**Figure 3:**
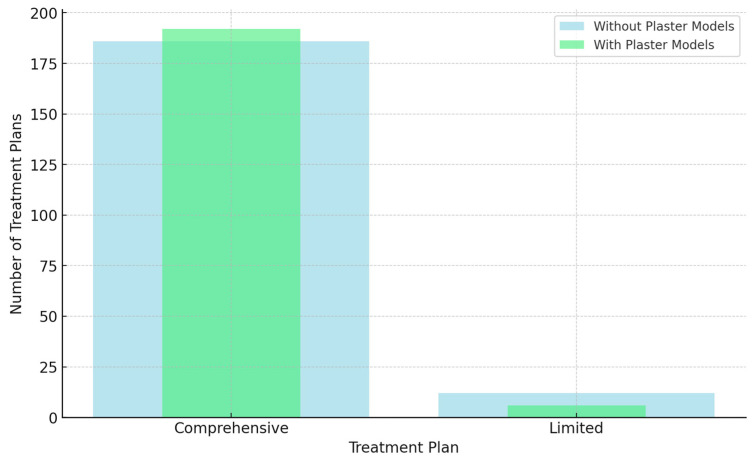
Impact of digital records vs. diagnostic plaster models on comprehensive vs. limited treatment plans.

Concerning choosing a comprehensive treatment plan vs. a limited treatment plan in treating the different studied malocclusion cases, for the first evaluation setting with digital records per se, the majority of treatment plans formulated by orthodontists (186 treatment plans) (93.9%) were reported by evaluating orthodontists to require comprehensive orthodontic intervention. At the same time, only 12 treatment plans (6.1%) were reported to require limited orthodontic treatment. For the second evaluation setting, aided with the addition of physical diagnostic plaster models, a minor increase in preference for comprehensive treatment was evident in 192 treatment plans (97%) and limited in 6 treatment plans (3%). However, this difference in treatment plan decisions based on digital records vs. physical models was statistically insignificant (*p* = 0.146), suggesting that plaster models did not significantly influence the orthodontists’ “Treatment Plan One” decision of comprehensive vs. limited treatment plans (Table 2; Figure 3).

#### 3.2.2. Treatment Plan Two (Surgical Intervention Treatment Plan vs. Non-Surgical Intervention Treatment Plan)

**Figure 4:**
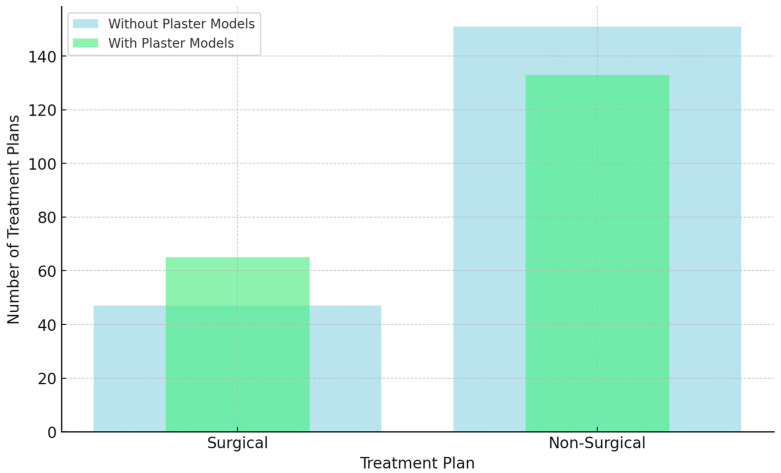
Impact of digital records vs. diagnostic plaster models on surgical vs. non-surgical treatment plans.

Regarding the need for surgical intervention in treatment planning for the different orthodontic malocclusion cases, in the treatment plans for the first evaluation setting with digital records per se, the majority of treatment plans formulated by orthodontists (151 treatment plans) (76.3%) were reported by evaluating orthodontists to be non-surgical, whereas surgical treatment plans were decided for 47 of the treatment plans (23.7%). For the second evaluation setting, with the addition of physical diagnostic plaster models, a statistically significant shift was reported in orthodontists’ treatment plans recommending surgical intervention for 65 treatment plans (32.8%) and non-surgical treatment plans 133 times (67.2%). This difference was statistically significant (*p* = 0.026), with a higher proportion of orthodontists advocating for surgery in the presence of physical plaster models (32.8%) compared to (23.7%) using digital records per se. This implied that plaster models did significantly influence the orthodontists’ “Treatment Plan Two” decision of surgical intervention vs. non-surgical intervention treatment plans (Table 2; Figure 4).

#### 3.2.3. Treatment Plan Three (Extraction vs. Non-Extraction)

**Figure 5:**
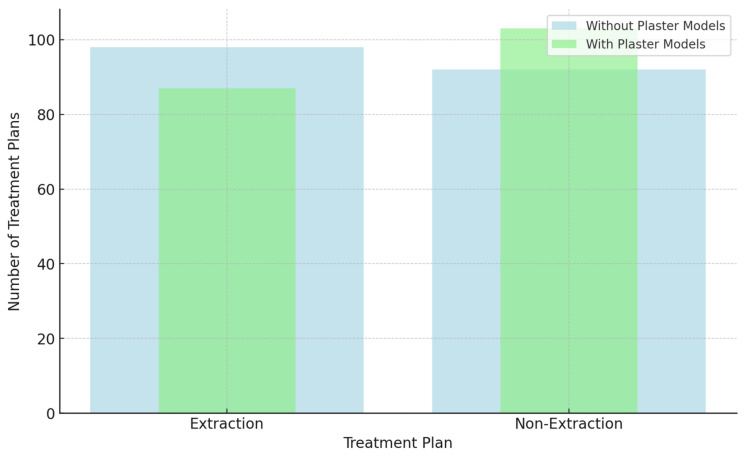
Impact of digital records vs. diagnostic plaster models on extraction vs. non-extraction treatment plans.

Concerning extraction vs. non-extraction treatment options for treating orthodontic malocclusion cases, for the first evaluation setting with digital records per se, extraction was chosen as a treatment option 98 times (51.6%), and non-extraction was chosen as a treatment option 92 times (48.4%), whereas for the second evaluation setting, with the addition of physical diagnostic plaster models, the distribution changed to extraction for 87 formulated treatment plans (45.8%), and non-extraction treatment plans were reported 103 times (54.2%). The slight decrease in preference for extractions (from 51.6% to 45.8%) as a result of adding the physical plaster diagnostic model to the digital records was not statistically significant (*p* = 0.266). This indicates that plaster models did not significantly influence the orthodontists’ “Treatment Plan Three” decision of interventional extraction vs. teeth preservation non-extraction treatment plans (Table 2; Figure 5).

## 4. Discussion

The effect of physical plaster orthodontic models on diagnosis and treatment planning has been a subject of ongoing research. The present study addressed the added value of physical plaster models offered to orthodontic diagnosis and treatment planning that primarily relied on digital records. This bridges a gap in the literature, as most previous research has centered on the potential of digital records substituting physical plaster models in diagnostic accuracy and treatment planning and not on the merit of having digital records plus physical diagnostic models.

Previous research has explored digital models as a substitute for plaster models in orthodontics, focusing on variations in diagnosis and treatment planning, the reliability and accuracy of measurements, and arch relationships [4,24,25,26]. Mixed results have been reported; some researchers advocated for digital records substituting physical records, whereas others expressed reservations about their generalized application. Several studies reported that no significant differences existed between digital records and plaster models with regard to the final treatment plans and reliability [3,13,19]. They concluded that digital models are a valid alternative for orthodontic diagnostic assessment. Similarly, other studies determined that digital records are accurate and realistic tools for documentation, analysis, treatment planning, and long-term follow-up in orthodontics and orthognathic surgery [8,22,27]. Likewise, another study reported that digital records did not lead to different malocclusion diagnoses compared to plaster models, supporting their use for treatment planning and diagnosis, and confirming that digital models are not a compromised choice for treatment planning or diagnosis [12]. However, other studies reported that digital records could replace plaster models in Class II malocclusion treatment planning, although evidence was insufficient to support their use for all other malocclusion types [4,21].

This study focused on the role of physical plaster models in orthodontic decision-making. The results of this study collectively reported on whether physical plaster models provided an added value as an adjunct tool in orthodontic diagnosis and treatment planning. It was reported that incorporating plaster models did not significantly alter the diagnosis of orthodontic malocclusion classification. On the other hand, integrating plaster models within the treatment planning phase led to a statistically significant shift towards recommending surgical intervention, suggesting their potential to reveal subtle skeletal discrepancies not readily apparent in digital records. Thus, it could be inferred that physical diagnostic orthodontic models offered valuable insights that can refine treatment strategies, particularly regarding surgical intervention in “Treatment Plan Two”. This enhanced understanding can ultimately lead to more personalized and effective treatment plans, thus improving the overall quality of orthodontic care.

The finding of this study revealed that plaster models did not significantly affect orthodontic malocclusion diagnosis nor “Treatment Plans One” or “Three”, suggesting that the value of including plaster models was evident in refining treatment specifics rather than dictating the overall scope of intervention. Nevertheless, it significantly impacted “Treatment Plan Two”, leading to an increased likelihood of surgical interventions due to the potential of plaster models revealing subtle skeletal discrepancies that may necessitate surgical intervention, thereby enriching the decision-making process in treatment planning.

The results of this study align with previous research demonstrating that relying on physical study models for orthodontics treatment planning provided sufficient information for treatment decisions in Class II malocclusion [2,6,28,29].

This was in opposition to other studies that reported no definitive evidence that dental models are needed for orthodontic diagnosis and treatment planning and confirmed that digital patient records could be an alternative to physical plaster models in assessing malocclusion and orthodontic treatment needs [13,23,29].

Other researchers could not confirm the complete validity of digital record replacement for physical models in orthodontic diagnosis and treatment planning decisions. One study suggested that digital dental records could replace conventional dental models for part of the clinical decision-making processes, though complete equivalency has yet to be established [4]. Equally, another study supported using digital models as an alternative to plaster models for assessing occlusal changes at the individual patient level but not fully at the group level for clinical audit or research purposes [30]. Despite the advancements and benefits of digital records, orthodontic plaster models retain unique characteristics that make them valuable, such as aiding in band size selection, visual communication, and serving as solid pretreatment records for medico-legal purposes [31].

This study’s findings suggest that while plaster models may influence orthodontists’ perception of the need for surgical intervention, they do not significantly impact the overall diagnostic classification nor the decision about the extent of treatment, whether comprehensive or limited, or whether the treatment needed extraction versus non-extraction approaches.

### Limitation

One of the limitations of this study is the non-equivalent inclusion of orthodontists with varying degrees of experience, which may have introduced some bias. Additionally, the sample size of orthodontists included in the study was relatively small. However, each orthodontist acted as their own control when evaluating treatment plans. Another limitation of this study is the inclusion of different degrees of malocclusion. Future studies should expand upon these findings by incorporating a larger, more diverse sample size of both orthodontists and malocclusion cases.

## 5. Conclusions

Within the limitations of this study, it can be concluded that the presence or absence of orthodontic plaster models does not significantly impact the orthodontic diagnosis or treatment planning process. By judiciously integrating plaster models into their diagnostic and treatment planning routine, orthodontists can have a more comprehensive understanding of their patient’s unique orthodontic needs, ultimately leading to more personalized and effective treatment outcomes. By recognizing the continuing value of conventional practices, such as the use of diagnostic plaster models, we can recommend that technological innovation such as digital records complement rather than replace the conventional diagnostic models.

While plaster models may not fundamentally alter the initial diagnosis, their ability to influence surgical treatment recommendations emphasizes their value as supplementary diagnostic tools. A comprehensive understanding of the strengths and limitations of different diagnostic aids, including both conventional and digital modalities, is crucial for optimizing patient care.

## Figures and Tables

**Table 1:** Distribution of participating orthodontists’ (*n* = 33) demographics.

Characteristics	No.(%)
Gender	
Male	23(69.7)
Female	10(30.3)
Professional position	
Consultant	21(63.6)
Specialists	12(36.4)
Area of practice	
Government	19(57.6)
Private	5(15.2)
Teaching institution	9(27.3)

**Table 2:** Comparison for diagnosis and treatment plans, without and with the presence of orthodontic plaster models.

	With Plaster Models							Total No. (%)	*p*-Value
	Outcome	Diagnosis							
		Class I		Class II		Class III			
Without Plaster Models	Diagnosis Class I	53	72.6%	16	24.2%	10	16.9%	79 (39.3)	<0.0001
		67.1%		20.3%		12.7%		100%	
	Class II	3	4.1%	49	74.2%	3	5.1%	55 (27.8)	
		5.5%		89.1%		5.5%		100%	
	Class III	17	23.3%	1	1.5%	46	78%	64 (32.3)	
		26.6%		1.6%		71.9%		100%	
	Total No. (%)	73 (36.9)	100%	66 (33.3)	100%	59 (29.8)	100%	198 (100)	
	Treatment 1 Comprehensive	Treatment 1						Total No. (%)	0.146
		Comprehensive			Limited				
		183		95.3%	3		50%	186 (93.9)	
		98.4%			1.6%			100%	
	Limited	9		4.7%	3		50%	12 (6.1)	
		75%			25%			100%	
	Total No. (%)	192 (97)		100%	6 (3)		100%	198 (100)	
	Treatment 2 Surgical	Treatment 2						Total No. (%)	0.026
		Surgical			Non-Surgical				
		27		41.5%	20		15%	47(23.7)	
		57.4%			42.6%			100%	
	Non-Surgical	38		58.5%	113		85%	151 (76.3)	
		25.2%			74.8%			100%	
	Total No. (%)	65 (32.8)		100%	133 (67.2)		100%	198 (100)	
	Treatment 3 Extraction	Treatment 3						Total	0.266
		Extraction			Non-Extraction				
		52		59.8%	46		44.7%	98 (51.6)	
		53.1%			46.9%			100%	
	Non-Extraction	35		40.2%	57		55.3%	92 (48.4)	
		38%			62%			100%	
	Total No. (%)	87 (45.8)		100%	103 (54.2)		100%	190 (100)	

## Data Availability

The data presented in this study are available on request from the corresponding author.
